# Serological Prevalence of *Schistosoma japonicum* in Mobile Populations in Previously Endemic but Now Non-Endemic Regions of China: A Systematic Review and Meta-Analysis

**DOI:** 10.1371/journal.pone.0128896

**Published:** 2015-06-04

**Authors:** Chao-Rong Bian, Da-Bing Lu, Jing Su, Xia Zhou, Hong-Xiang Zhuge, Poppy H. L. Lamberton

**Affiliations:** 1 Department of Epidemiology and Statistics, School of Public Health, Soochow University, Suzhou 215123, China; 2 Department of Parasitology, School of Biology and Basic Medical Sciences, Soochow University, Suzhou 215123, China; 3 Department of Infectious Disease Epidemiology, Imperial College London, London, W2 1PG, United Kingdom; The George Washington University School of Medicine and Health Sciences, UNITED STATES

## Abstract

**Background:**

Schistosomiasis japonica has been resurging in certain areas of China where its transmission was previously well controlled or interrupted. Several factors may be contributing to this, including mobile populations, which if infected, may spread the disease. A wide range of estimates have been published for *S*. *japonicum* infections in mobile populations, and a synthesis of these data will elucidate the relative risk presented from these groups.

**Methods:**

A literature search for publications up to Oct 31, 2014 on *S*. *japonicum* infection in mobile populations in previously endemic but now non-endemic regions was conducted using four bibliographic databases: China National Knowledge Infrastructure, WanFang, VIP Chinese Journal Databases, and PubMed. A meta-analysis was conducted by pooling one arm binary data with MetaAnalyst Beta 3.13. The protocol is available on PROSPERO (No. CRD42013005967).

**Results:**

A total of 41 studies in Chinese met the inclusion criteria, covering seven provinces of China. The time of post-interruption surveillance ranged from the first year to the 31^st^ year. After employing a random-effects model, from 1992 to 2013 the pooled seroprevalence ranged from 0.9% (95% CI: 0.5-1.6%) in 2003 to 2.3% (95% CI: 1.5-3.4) in 1995; from the first year after the disease had been interrupted to the 31^st^ year, the pooled seroprevalence ranged from 0.6% (95% CI: 0.2-2.1%) in the 27th year to 4.0% (95%CI: 1.3-11.3%) in the second year. The pooled seroprevalence in mobile populations each year was significantly lower than among the residents of endemic regions, whilst four papers reported a lower level of infection in the mobile populations than in the local residents out of only 13 papers which included this data.

**Conclusions:**

The re-emergence of *S*. *japonicum* in areas which had previously interrupted transmission might be due to other factors, although risk from re-introduction from mobile populations could not be excluded.

## Introduction

Schistosomiasis, caused by infection with *Schistosoma* spp. including *Schistosoma haematobium*, *S*. *intercalatum*, *S*. *japonicum*, *S*. *mansoni*, and *S*. *mekongi*, is the second most important parasitic disease after *Plasmodium* in causing severe morbidity to humans in tropical and subtropical regions, with an annual loss of more than 70 million disability adjusted life years (DALYs) [1]. In China, over the past six decades, great success has been achieved in controlling schistosomiasis japonica. For example, the disease has been eradicated in five out of 12 provinces and transmission has been interrupted in 274 out of 454 counties (city or districts) [2]. However, schistosomiasis remains a major public health problem in China today, with over 245 million people living and 0.29 million people infected in 180 counties which are still endemic for the parasite [2]. Of major concern is that the disease has been resurging in previously well controlled or interrupted (i.e. post-interruption) areas [3,4]. Although several potential factors have been proposed, one main concern is with regard to the increasing number of mobile people who come from endemic areas but work or live in such post-interruption areas for a short period of time. To minimize re-emergence in currently controlled areas, it is paramount that the cause of such re-introduction be fully elucidated. This will then enable public health policy makers to focus on cost-effective surveillance and control strategies, and maximize China’s success in reducing human schistosomiasis across the entire country.

In China, in 1998, the size of the mobile population was estimated to be 156 million, out of which at least 30 million came from schistosome endemic areas [5]. By 2012, the mobile population had increased to approximately 236 million [6]. Infections with schistosomes among mobile people have been increasingly reported, for example, from 2005 to 2008 a total of 911 cases of acute schistosomiasis were reported nation-wide, out of which 60 (6.59%) cases were identified as non-local residents (i.e. in mobile populations who were currently working and/or living in a region from where they did not originate) [7]. Such imported schistosomiasis were also frequently reported outside China due to people traveling abroad [8,9]. This level of infection in mobile populations may be a key driving force affecting the spread or resurgence of schistosomiasis, especially in the areas or regions where schistosomiasis transmission had previously been interrupted but where the intermediate host snails remain present. The presence of snails is necessary for the parasite to complete its life cycle, with a compulsory asexual stage of development, and therefore for endemic transmission to potentially reoccur.

Due to these potential risk factors posed by mobile populations post-interruption surveillance for schistosome infections in regional immigrants is, therefore, of great importance if they are significantly involved in driving reinvasions of *S*. *japonicum* transmission. Due to differences among studies in year, period post-interruption or serological assays employed in the surveillance for schistosome infection among mobile people, a wide range of estimates have been reported. For example, in 1998 no infections (0/3622) were identified in the mobile population in Nanhui district of Shanghai [10]; whereas in 2008 up to 22.0% (87/396) of people were positive in the mobile population in Changshan county of Zhejiang province [11]. As their full significance remains to be elucidated, a synthesis of these seroprevalence data is needed. We have conducted a meta-analysis of previously published data to establish an improved estimate of the prevalence of schistosome infection in mobile populations currently working and/or residing in previously endemic but now non-endemic regions, and to investigate whether the seroprevalence has decreased over time or with year post-interruption, due to continued success in reducing transmission in endemic areas [2]. This has implications for public policy making and planning in the assessment of the relative influence that such mobile populations have on re-emergence of the parasite in the post-interruption areas, versus other potential factors, and where control strategies and surveillance will be best deployed.

## Methods

### Search strategy and selection criteria

A literature search for publications up to Oct 31, 2014 on *S*. *japonicum* infection in mobile populations in previously endemic but now non-endemic areas or regions (i.e. counties or districts) was conducted using the four bibiliographic databases (three in Chinese and one in English): China National Knowledge Infrastructure (CNKI), WanFang, VIP Chinese Journal Databases, and PubMed. We used the terms ‘(liudong [mobile or floating] or shuru or wailai [imported]) and xuexichong [schistosome]’ in Pinyin (phoneticism) in Chinese databases and the terms ‘schisto* and (surveillance or monitor or investigation or prevalence) and (interrupt* or eliminat*) and China’ in the English database (PubMed) in our searches. The criteria for transmission interruption in China in previously endemic areas are defined as 1) prevalence of human and/or cattle infections less than 0.2% by parasitological examination; 2) no new infection of human and/or cattle found for five consecutive years; 3) no snails found for more than one year (or no infected snails found in marshlands) [3]. A mobile population is defined as the people who are not local residents but have worked and/or lived there for at least a month. The reference lists of relevant reviews and articles were also examined. We limited the language of the studies to English or Chinese.

All search results were limited to observational studies conducted on mobile populations. Studies had to meet the following criteria for inclusion: they had to report the number of schistosome infections, the number of participants who had been tested and the year of surveillance. For a longitudinal study, the number of infections and the number of participants must have been reported within each year, for data from that specific year to have been included. Due to low sensitivities of parasitological techniques, particularly as intensities reduced with continued treatment success [12], the infection had to be detected with serological tests. Studies were excluded if the number of mobile people was not reported separately from the number of the local residents and could not be obtained from the authors; if the methods were unclear; or if the studies were reviews, local or government reports, conference abstracts or presentations, or degree theses.

### Choice of inclusion criteria for serological tests

A series of immunodiagnostic tests, including Indirect Hemagglutination Assay (IHA), Enzyme Linked Immunosorbent Assay (ELISA), Dot Immuno-Gold Filtration Assay (DIGFA), Circum Oval Precipitation Test (COPT), Intradermal Test (ID), Dye Dipstick Immuno-Assay (DDIA) and Immuno-Enzymatic Staining Test (IEST), have been used as a surveillance tool. ID is a simple approach and had shown a high sensitivity among patients identified through egg detection, but the result may remain positive for several years after patients had been effectively treated [13]. COPT had been proven to be both sensitive and specific. However, its sensitivity declined rapidly when the infection prevalence and intensity of infection were significantly lower [14]. These have now been replaced with IHA, ELISA or other advanced methods. Previous studies suggested that ELISA could be useful in *S*. *japonicum* diagnosis in low or moderate endemic regions [15], and IHA could be considered as a surveillance approach for verifying elimination of schistosomiasis [16]. IEST was shown to be more sensitive and specific than COPT [17]. Developed for field application, DDIA is a rapid and simple tool with both high sensitivity and adequate specificity [18,19], even in low prevalence and previously-treated populations [20]. DIGFA presents a similar sensitivity and specificity as ELISA or IHA, and is also suitable for large-scale application as no specific instrument is required [21]. Although the above serological tests cannot identify active infections as direct parasitological methods, they indeed have shown the merits of high sensitivity, ease of use and rapidity. The important role and usefulness of such immunodiagnostics in the screening for schistosomiasis or surveillance has been discussed elsewhere [22]. We included studies which utilized any of these above serological tests.

We evaluated the risk of bias among the included studies using a quality assessment checklist. The following items were examined and each given a score based on a simple scale system (see notes in Table 1): i) was the research question/objective clearly described and stated? ii) was the mobile population clearly defined? iii) was the infection of *S*. *japonicum* measured with a valid serological antibody test? iv) was the endemic region from where the mobile population originated clearly described? v) were more than 50 subjects in total included in the study?

**Table 1:** Study characteristics and data summaries of the included publications.

							Quality assessment				
Author, year	County (city or district), Province	Year* when interrupted	Study period	Snails found**	Sero prevalence in local residents (sample size)	Sero prevalence in mobile population (sample size)	Research question	Definition of mobile population	Serological test†	Proportion from endemic areas^‡^	Score^§^
Fang et al, 2009	Huangshan, Anhui	1993	2007	Yes	0.25% (3576)	0.47% (422)	Clear	Clear	IHA	24.9%	10
Yan et al, 2012	Longhai, Fujian	1985	1989–2010	Yes	0–3.4% in children aged 7–14 years (41–328)	0 (102; 410)	Clear	Quite clear	IHA	Unknown	8
Xu et al, 2013	Huadu and Zengcheng, Guangdong	1982	2006–2010	No	0% (200–600)	0–3.17% (200–1027)	Clear	Clear	DIGFA	Unknown	9
Li et al, 2012	Qingxin, Guangdong	1984	2006–2010	No	0–4.2% in children (215–307)	0.67–6.19% (100–1002)	Clear	Quite clear	DIGFA	Unknown	8
Gao et al, 2003	Zengcheng and Huadu, Guangdong	1982	1996–2000	No	2.3–4.8% in children (353–393)	2.22–5.88% (45–102)	Clear	Clear	ID	Unknown	8
Gao et al, 2007	Zengcheng and Huadu, Guangdong	1982	2001–2005	No	0% in children (200–209)	2.08–5.88% (48–102)	Clear	Clear	ELISA	Unknown	8
Zhou et al, 2008	Shaoguan, Guangdong	1985	2001–2006	No	0% in children (387–487)	2.30–5.88% (51–98)	Clear	Clear	ELISA	Unknown	9
Su et al, 2014	Zhaoqing, Guangdong	1985	2008–2013	No	0.25–0.61% (313–595)	1.16–1.84% (345–762)	Clear	Clear	DIGFA	AH 40.4%, HB 20.4%, HN 10%, JS 11.9%, JX 20.1%, SC 6.5%, YN 0.8%	10
Lin et al, 2009	Counties, Guangxi	1988	2008	Yes		3.46% (2428)	Clear	Clear	IHA	48.2%	10
Zhou et al, 2009	Changshu, Jiangsu	1990	2008	No	0.47% (1066)	0 (124)	Clear	Clear	DDIA	AH 57.3%, SC 6.5%, JS 25.8%	10
Wu et al, 1994	Changshu, Jiangsu	1990	1992	Unclear	0.1% (21421)	6.87% (9759)	Clear	Clear	IEST	AH 60%, SC 4%	10
Xue et al, 1997	Jiangyin, Jiangsu	1981	1995	Unclear		3.75% (1067)	Clear	Clear	IEST	AH 36.6%, SC 16.5%	10
Zhou et al, 2011	Tongzhou, Jiangsu	1993	2002–2009	No	0.49–0.5% in children (200–205)	0.79–1.45% (207–312)	Clear	Clear	DDIA	Unknown	9
Ji et al, 2000	Tongzhou, Jiangsu	1993	1996–1999	No	0.85–1.81% in children (332–468)	0.95–5.65% (248–446)	Clear	Clear	ID	Unknown	10
Jin et al, 2006	Jinshan, Shanghai	1984	2005	Yes	0.59% (507)	0 (103); 0.06% (11690)	Clear	Clear	IHA/COPT	38.30%	9
Yang et al, 2007	Jinshan, Shanghai	1984	2006	Yes	4.47% (514)	0.06% (12747); 3.56% (505)	Clear	Clear	IHA/IEST	Unknown	8
Yang et al, 2008	Jinshan, Shanghai	1984	2007	No	1% (501)	0.05% (14300); 0.60% (504)	Clear	Clear	IHA/IEST	Unknown	8
Yu et al, 2009	Jinshan, Shanghai	1984	2008	Yes		1.66% (602)	Clear	Clear	IHA	All	10
Zhou et al, 2007	Minhang and Pudong, Shanghai	1985	2004	Yes		4.71% (2931)	Clear	Clear	IHA	66.12%	10
Shi et al, 2012	Minhang, Shanghai	1985	1994–2009	Unclear		0.05–5.37% (8325–37393)	Clear	Clear	IEST	AH 37.2%, SC 18.5%, JS 12.2, HN 8.2%	10
He et al, 2002	Jinshan, Shanghai	1984	1999	Unclear		4.45% (1281)	Clear	Clear	IEST	63.90%	10
Song et al, 2011	Nanhui, Shanghai	1985	2000–2009	Yes		0.13–1.01% (3671–11019)	Clear	Clear	IEST	AH 28.8%, JS 19.2%, ZJ 12.1%, SC 9.1%, JX 10%, HN 6.2%	10
Jin et al, 2010	Minhang, Pudong, Jiading, Songjiang and Jinshan, Shanghai	1985	2008	Yes		2.14% (2992)	Clear	Clear	IHA	All	10
Qiu et al, 2010	Luwan, Shanghai	1985	1995–2008	Unclear		0.10–2.32% (725–4635)	Clear	Clear	IEST	Unknown	9
Song et al, 2005	Nanhui, Shanghai	1985	1994–2003	Yes		0–0.67% (2687–8487)	Clear	Clear	IEST	AH 27.9%, JS 18%, ZJ 12.3%, SC 8.6%, JX 9.8%, HN 6.8%	10
He et al, 2006	Qingpu, Shanghai	1983	1995–2004	No		1.20–3.52% (5247–34228)	Clear	Clear	IEST	Unknown	9
Dang et al, 2005	Jinshan, Shanghai	1984	2000–2002	Yes		0.32% (313)	Clear	Clear	DIGFA/ELISA	AH 53.7%, JS 16%	9
Li et al, 1996	Baoshan, Shanghai	1984	1993	Unclear		5.61% (4809)	Clear	Clear	ID	AH 12.4%, JS 13.6%, ZJ 10.6%, SC 13.1%, JX 8%, HN 9.3%	10
Yuan et al, 2002	Baoshan, Shanghai	1984	2000	Unclear		0.56% (4797)	Clear	Clear	IEST	AH 28%, JS 23.6%, SC 14%	10
Zhang et al, 2011	Changshan, Zhejiang	1995	2005–2009	Yes	0.2–8.12% (501–530)	0.85–21.97% (103–896)	Clear	Clear	IHA	Unknown	9
Li et al, 2007	Yuhang, Zhejiang	1994	2006	Unclear		0.33% (3307)	Clear	Clear	DIGFA	73.80%	10
Xie et al, 2010	Zhuji, Zhejiang	1994	2008–2009	Unclear		3.08% (1979); 5.33% (900)	Clear	Clear	IHA	All	10
Zhu et al, 2012	Jiaxing, Zhejiang	1993	2008–2011	Yes	0.48–5.07%	0.16–6.70% (723–2443)	Clear	Clear	IHA	Unknown	9
Chen et al, 2008	Cixi, Zhejiang	1981	2004–2007	Unclear		1.60–5.22% (690–13295)	Clear	Clear	IHA	Unknown	9
Xu et al, 2009	Cixi, Zhejiang	1981	2007–2008	No		0.26% (12330)	Clear	Clear	DIGFA	Unknown	9
Hu et al, 2014	Cixi, Zhejiang	1981	2008–2012	No	0–0.86% (200–296)	0.99–2.98% (300–507)	Clear	Clear	IHA	Unknown	9
Xu et al, 2012	Changshan, Zhejiang	1995	2008–2011	Yes	1.21–4.79% (330–501)	0.81–10.00% (124–536)	Clear	Clear	IHA	Unknown	9
Wang et al, 2013	Jiaxing, Zhejiang	1994	1995–2012	Yes	0.2–2.6% (283–50257)	0.67–4.85% (9063–28050)	Clear	Clear	IHA/DIGFA/COPT/ELISA	Unknown	8
Zhou et al, 1998	Jiaxing, Zhejiang	1994	1997	Yes		5.24% (3361)	Clear	Clear	IEST	62%	10
Xu et al, 2009	Yinzhou, Zhejiang	1985	2006	Unclear		0.73% (19403)	Clear	Clear	IEST	AH 30.9%, JX 26.9%, JS 3.1%, SC 21.1%, HN 7.8%, HB9.2%, YN 1%	10
Lou et al, 2001	Yiwu, Zhejiang	1994	1999	Unclear		1.88% (849)	Clear	Clear	DIGFA	Unknown	9

Note:

* At the level of county (city or district);

** Snails were found in the year or at least at the end of study period;

^†^ IHA, Indirect Hemagglutination Assay; DIGFA, Dot Immuno-Gold Filtration Assay; ID, Intradermal Test; ELISA, Enzyme Linked Immunosorbent Assay; DDIA, Dye Dipstick Immuno-Assay; IEST, Immuno-Enzymatic Staining Test; COPT, Circum Oval Precipitation Test.

^‡^ AH, Anhui province; SC, Sichuan; JS, Jiangsu; JX, Jiangxi; HN, Hunan; HB, Hubei; ZJ, Zhejiang; YN, Yunnan.

^§^ Each item was scaled as 1 or 2: research question ('unclear' 1 or 'clear' 2); definition of the mobile population ('unclear' 1 or 'clear' 2); serological test (''two or more tests' 1, or 'one test' 2); origin of the mobile population in endemic areas (unknown proportion 1, or known proportion 2); and sample size of mobile population ('less than 50' 1 or 'equal or more than 50' 2).

Screening of the initial search results (i.e. titles and abstracts) was performed independently by two reviewers for each abstract (CRB and JS). Any discrepancies on exclusions were discussed and mutually resolved. After the first exclusion according to the criteria described above, the full manuscripts of the remaining articles were each screened independently by the two reviewers. Any discrepancies between the reviewers were discussed and a mutual agreement made on whether each manuscript met all inclusion criteria.

### Data extraction

Two reviewers independently extracted the characteristics of each included study onto pre-designed Excel forms. These included publication year, authors, study participant eligibility criteria, study period, numbers of individuals positive for *S*. *japonicum*, the total number of individuals tested, and the serological test used. Any discrepancy in data extraction was resolved by consensus and consulting a third reviewer if necessary.

### Strategy for data synthesis

A meta-analysis of the seroprevalence of *S*. *japonicum* in mobile populations was conducted by pooling one arm binary data using MetaAnalyst Beta 3.13 [23]. Since infections in residents in endemic areas of China have been greatly reduced, for example from 11.61 million in 1950s [24], to 0.84 million in 2003 [24] and to 0.29 million in 2011 [2], we here pooled the seroprevalence in mobile populations in post-interruption areas each year to see if there was also a decreasing trend over time. After transmission of the disease has been interrupted, and if this achievement has been consolidated, then it may also be inferred that the chances of immigrant populations to become infected, from either their endemic origin or their current local region, should also decrease over time. We then pooled the seroprevalence in mobile populations of such areas in each year by time post-interruption.

We estimated heterogeneity between studies within each year (or in each period post-interruption) with Cochran’s Q, a statistic based on the chi-squared test, and the *I* ^2^ statistic, which describes the percentage of variation between studies that is due to heterogeneity rather than chance [25]. Differing from Q, the index of *I* ^2^ does not rely on the number of studies included, with values of 25%, 50% and 75% indicating low, moderate, and high degrees of heterogeneity, respectively. If the value of *I* ^2^ is less than 50%, we use a fixed-effects model to calculate the point estimate of seroprevalence and its 95% confidence interval (CI). Publication bias of studies was statistically examined with the Begger test [26,27] in Stata/SE (version 11.2). In addition, the estimate of the pooled seroprevalence in mobile populations was also compared with the seroprevalence in residents from currently endemic regions from the national surveillance program of China [2,28,29,30,31,32,33,34,35].

Analysis was in accordance with the preferred reporting items (see S1 Table) for systematic reviews and meta-analyses (PRISMA) guidelines [36], and the protocol has been previously registered in PROSPERO [37] which is available on http://www.crd.york.ac.uk/PROSPERO/display_record.asp?ID=CRD42013005967. No ethical approval was needed for this research because all data used are secondary summary data.

## Results

**Fig 1:**
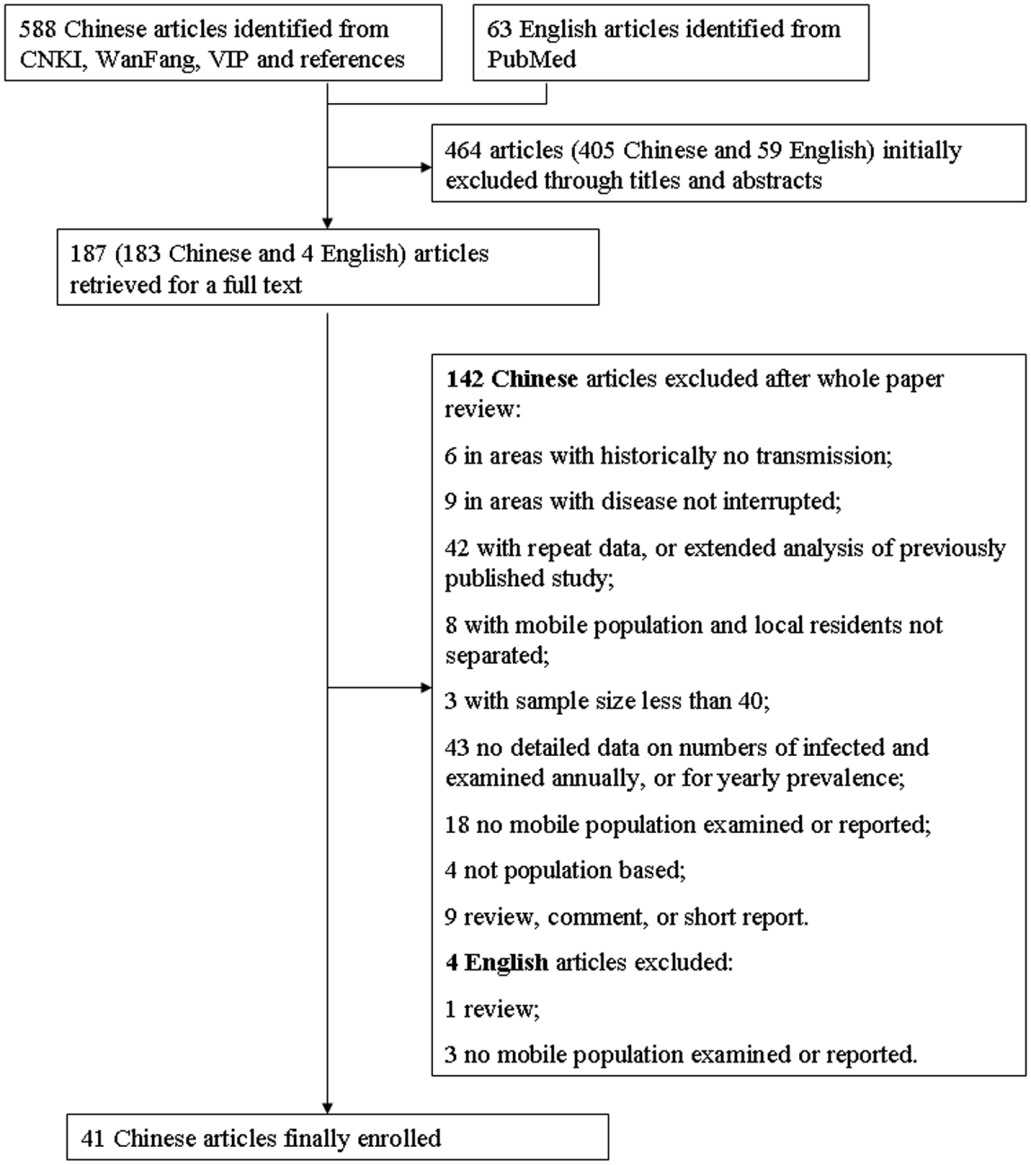
The flow diagram of paper review process. One article may contain one or more studies.

Our searches across all four databases returned a total of 651 records with 588 in Chinese and 63 in English. After removal of duplicates and initial screening through titles and abstracts, we reviewed 187 papers in full with 183 in Chinese and four in English (Fig 1). The four English articles were subsequently excluded because: one is a short review on the experiences of elimination of schistosomiasis in Shanghai municipality [38]; two mentioned the surveillance for the disease in the mobile population, but with no seroprevalence data [39] or no seroprevalence data by year [40]; and one did not involve any surveillance work in the mobile population [41]. Of 183 articles in Chinese, 142 were ineligible and then excluded. The reasons include duplication (apparent in the full text, but not from the abstract alone), lack of detailed data on mobile populations, studies not conducted in post-interruption areas, sample size <50 individuals and articles being reviews and not research papers (for details see Fig 1). Our final sample was 41 studies published between August 1996 and May 2014. The included studies had been based in seven provinces of China, Anhui [42], Fujian [43], Guangdong [44,45,46,47,48,49], Guangxi [50], Jiangsu [51,52,53,54,55], Shanghai [10,56,57,58,59,60,61,62,63,64,65,66,67,68,69], and Zhejiang [11,70,71,72,73,74,75,76,77,78,79,80]. In addition to the compulsory inclusion criteria, 16 presented that snails capable of transmitting *S*. *japonicum* were found in the associated areas to where the mobile population had moved; 21 reported the proportion of the mobile population that had originated from endemic areas; and 13 reported the serological prevalence for *S*. *japonicum* in local residents in the areas with transmission interrupted, with four showing a level of infection that is lower than in the mobile population. The start of post-interruption surveillance ranged from the first year after up to the 31^st^ year after disease transmission had been interrupted. The characteristics of each research paper included, the seroprevalence or its range in populations, and the score of each report based on the simple quality assessment are detailed in Table 1 (the detailed prevalence in mobile populations by study and year are provided in S2 Table).

**Fig 2:**
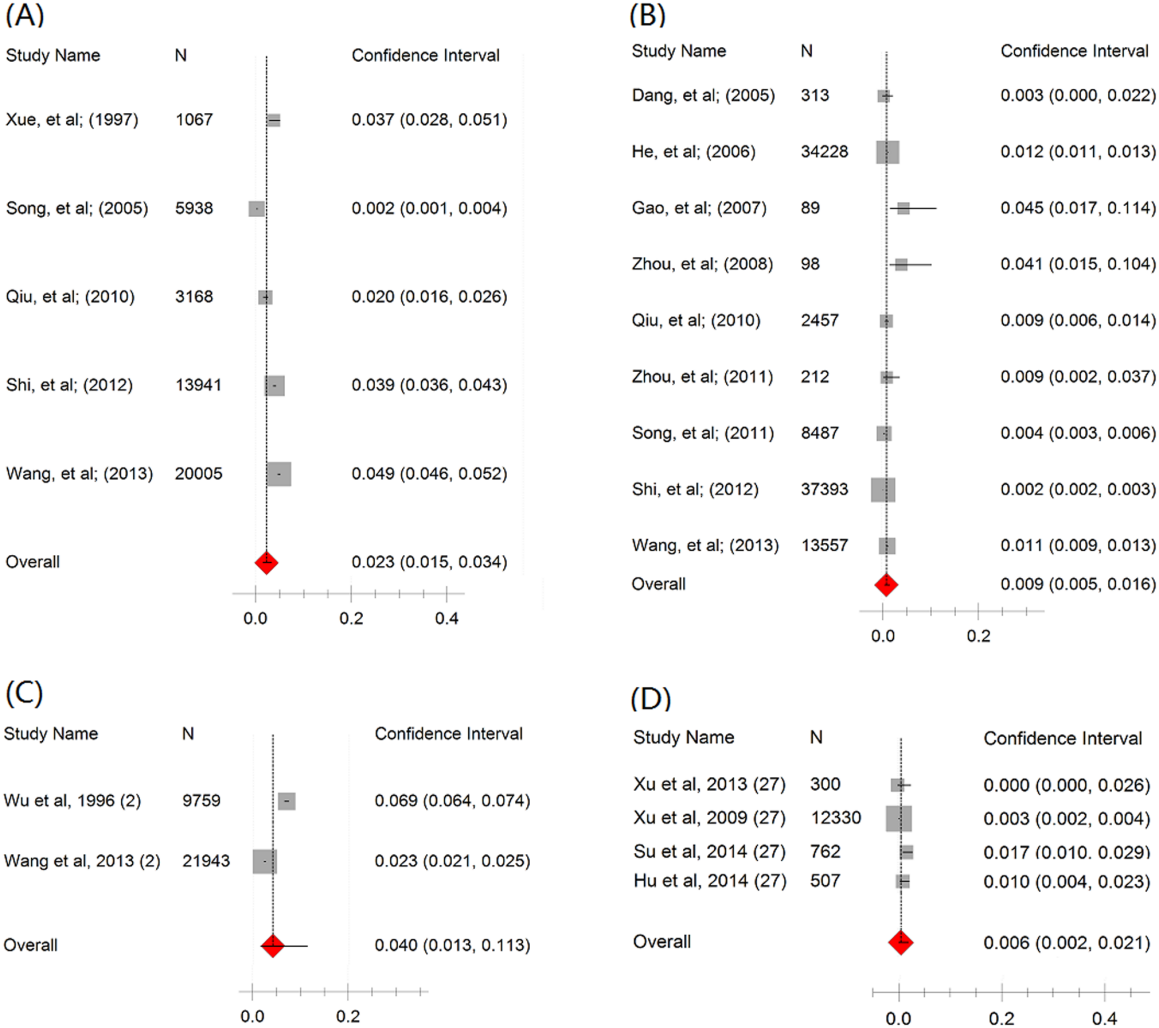
Forest plot of seroprevalence in mobile populations based on a random-effects model. A in 1995, B in 2003, C in the 2^nd^ year post-interruption and D in the 27^th^ year post-interruption.

**Fig 3:**
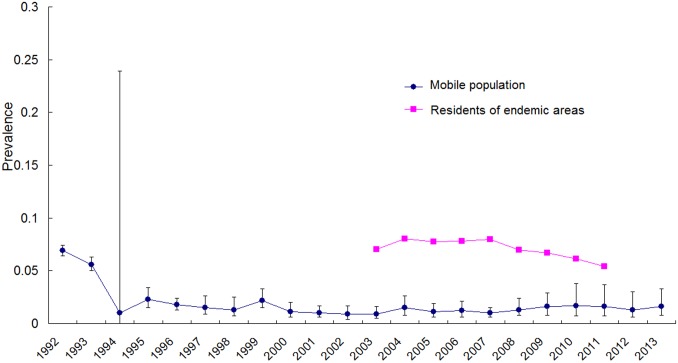
The pooled seroprevalence of schistosome infection in mobile populations and residents of endemic areas by year. Weights are from random-effects analysis.

**Fig 4:**
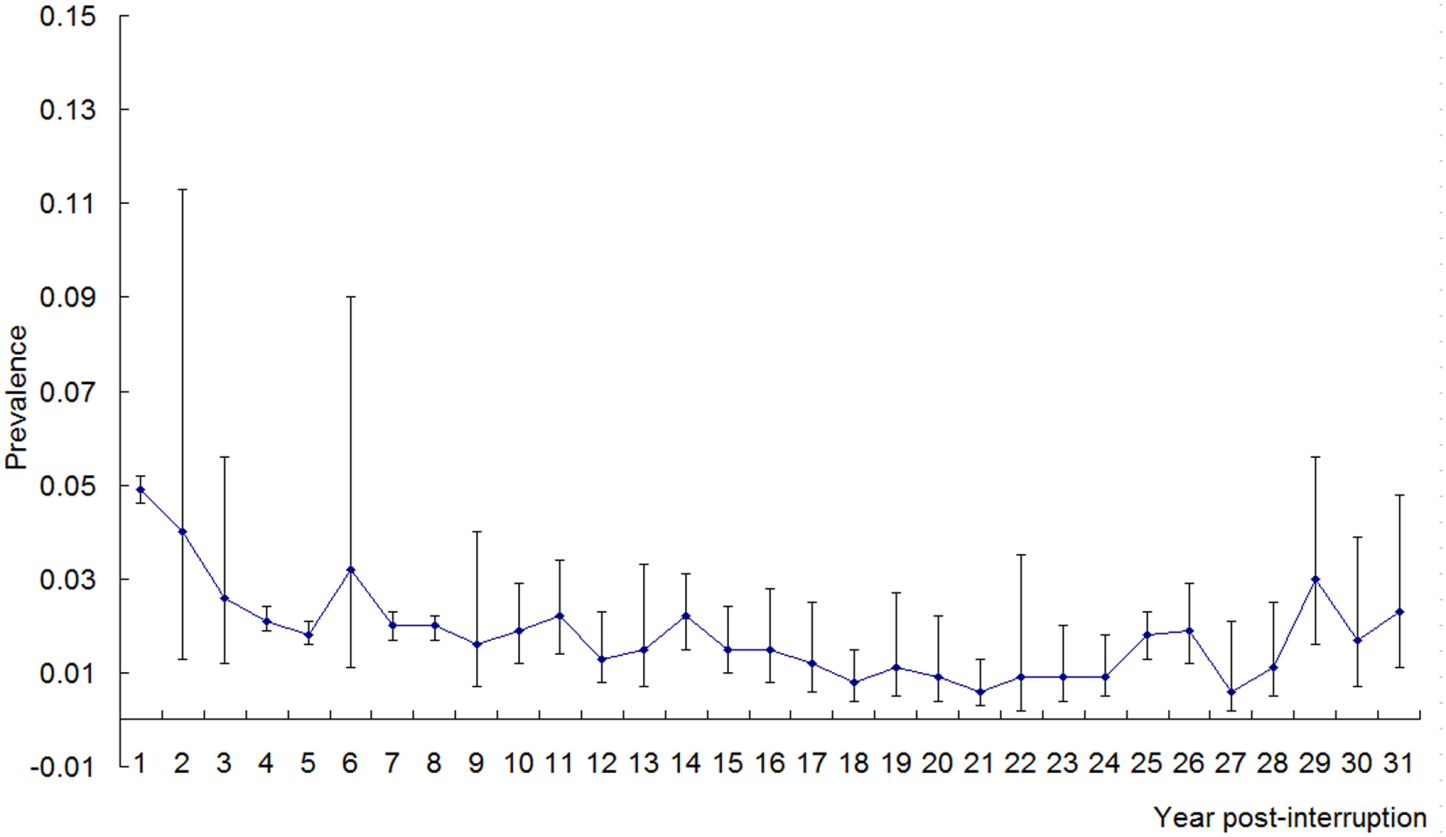
The pooled seroprevalence of schistosome infection in mobile populations by year post-interruption. Weights are from random-effects analysis.

**Fig 5:**
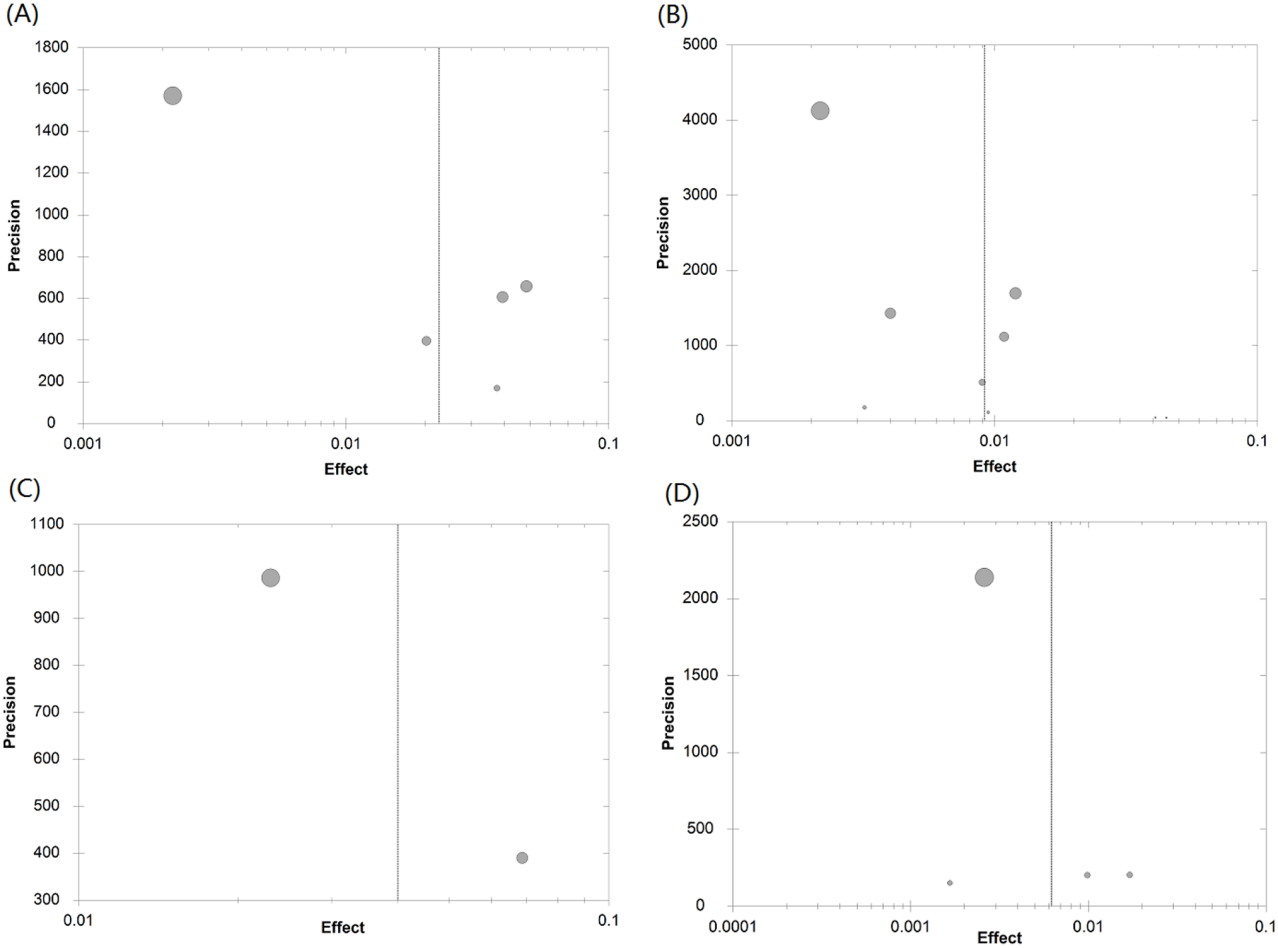
Publication bias plot. A in 1995, B in 2003, C in the 2^nd^ year post-interruption and D in the 27^th^ year post-interruption.

As shown in Tables 2 and 3, there was a lack of consistency between the two tests (Cochran’s Q and I^2^) for heterogeneity. Statistics Q indicated a random-effects model should be applied in the majority of analyses, whereas I^2^ indicated a fixed-effects model in all analyses. Therefore, we calculated the pooled seroprevalence of the included studies using both models (see Tables 2 and 3). After employing a random-effects model and excluding the year in which there was only one study, from 1992 to 2013 the estimated seroprevalence in mobile populations ranged from 0.9% (95% CI: 0.5–1.6%) in 2003 to 2.3% (95% CI: 1.5–3.4) in 1995 (Table 2). From the first year after the disease had been interrupted to the 31^st^ year, the estimated seroprevalence ranged from 0.6% (95% CI: 0.2–2.1%) in the 27^th^ year to 4.0% (95%CI: 1.3–11.3%) in the second year (Table 3). After employing a fixed-effects model, from 1992 to 2013, the estimated seroprevalence ranged from 0.8% (95% CI: 0.7–0.9%) in 2012 to 5.0% (95% CI: 4.5–5.4%) in 1994; within the period of 31 years post-transmission interrupted, the estimated ranged from 0.5% (95% CI: 0.4–0.6%) in the 27^th^ year to 4.3% (95%CI: 4.0–4.5%) in the second year (see Tables 2 and 3). Fig 2 shows the forest plot of the pooled seroprevalence, based on a random effects model, in 1995 and 2003, and in the 2^nd^ and the 27^th^ year post-interruption. Generally, the pooled seroprevalence of *S*. *japonicum* in the mobile population decreased over time (for a random-effects model, r_s_ = -0.219, P = 0.33; and for a fixed-effect model r_s_ = -0.824, P<0.01) and has remained at a very low level since 1994. Fig 3 shows the time trend of the seroprevalence in mobile populations by year, again remaining low from 1994 onwards. As seen in Fig 4, the estimated seroprevalence in the mobile populations also seemed to slowly but significantly decrease with year post-interruption (for a random-effects model, r_s_ = -0.491, P<0.01; and for a fixed-effect model, r_s_ = -0.507, P<0.01). Fig 5 presents the precision funnel plots of studies performed at the four time points mentioned above, with no clear effect in 1995 (5A), 2003 (5B), in the 2^nd^ year post-interruption (5C) nor in the 27^th^ year post-interruption (5D).

**Table 2:** Heterogeneity and pooled seroprevalence (%, 95% CI) of studies among mobile populations by year from 1992 to 2013.

							Random-effects			Fixed-effects		
Time	No. studies	Sample size	I^2^	Q	DF	P-val (Q)	Prev.	Lower	Upper	Prev.	Lower	Upper
1992	1	9759	NA	NA	NA	NA	0.069	0.064	0.074	0.069	0.064	0.074
1993	1	4809	NA	NA	NA	NA	0.056	0.05	0.063	0.056	0.05	0.063
1994	2	14385	0.498	0.992	0.5	<0.001	0.01	0	0.239	0.05	0.045	0.054
1995	5	44119	0.494	0.994	0.8	<0.001	0.023	0.015	0.034	0.042	0.04	0.044
1996	7	49440	0.477	0.985	0.857	<0.001	0.018	0.013	0.024	0.021	0.02	0.023
1997	8	52916	0.495	0.997	0.875	<0.001	0.015	0.009	0.026	0.021	0.02	0.023
1998	7	42942	0.495	0.996	0.857	<0.001	0.013	0.007	0.025	0.022	0.02	0.024
1999	9	47210	0.492	0.996	0.889	<0.001	0.022	0.015	0.033	0.025	0.023	0.026
2000	7	52797	0.495	0.996	0.857	<0.001	0.011	0.006	0.02	0.02	0.019	0.022
2001	8	51439	0.488	0.993	0.875	<0.001	0.01	0.006	0.017	0.014	0.013	0.015
2002	7	55360	0.495	0.997	0.857	<0.001	0.009	0.004	0.017	0.021	0.02	0.023
2003	9	96834	0.492	0.996	0.889	<0.001	0.009	0.005	0.016	0.009	0.009	0.01
2004	10	79104	0.496	0.998	0.9	<0.001	0.015	0.008	0.026	0.018	0.016	0.019
2005	13	87443	0.494	0.998	0.923	<0.001	0.011	0.006	0.019	0.015	0.014	0.016
2006	16	106458	0.496	0.999	0.933	<0.001	0.012	0.006	0.021	0.014	0.014	0.016
2007	14	79182	0.49	0.997	0.929	<0.001	0.01	0.006	0.015	0.019	0.018	0.02
2008	20	79612	0.495	0.999	0.95	<0.001	0.013	0.008	0.024	0.02	0.019	0.022
2009	13	47630	0.493	0.998	0.923	<0.001	0.016	0.008	0.029	0.018	0.017	0.019
2010	9	28452	0.489	0.995	0.889	<0.001	0.017	0.007	0.038	0.012	0.011	0.014
2011	7	19365	0.483	0.989	0.857	<0.001	0.016	0.007	0.037	0.011	0.01	0.013
2012	3	17904	0.473	0.951	0.667	<0.001	0.013	0.006	0.03	0.008	0.007	0.009
2013	1	443	NA	NA	NA	NA	0.016	0.008	0.033	0.016	0.008	0.033

**Table 3:** Heterogeneity and pooled seroprevalence (%, 95% CI) of studies among mobile populations by year post-transmission interruption.

							Random-effects			Fixed-effects		
Year post-interruption	No. studies	Sample size	I^2^	Q	DF	P-val (Q)	Prev.	Lower	Upper	Prev.	Lower	Upper
1	1	20005	NA	NA	NA	NA	0.049	0.046	0.052	0.049	0.046	0.052
2	2	31702	0.499	0.997	0.5	<0.001	0.04	0.013	0.113	0.043	0.04	0.045
3	3	19446	0.495	0.99	0.667	<0.001	0.026	0.012	0.056	0.029	0.026	0.031
4	2	12605	0	0.014	0.5	0.475	0.021	0.019	0.024	0.021	0.019	0.024
5	3	13921	0	0.575	0.667	0.337	0.018	0.016	0.021	0.018	0.016	0.021
6	2	10873	0.483	0.939	0.5	<0.001	0.032	0.011	0.09	0.021	0.018	0.024
7	1	9063	NA	NA	NA	NA	0.02	0.017	0.023	0.02	0.017	0.023
8	1	10753	NA	NA	NA	NA	0.02	0.017	0.022	0.02	0.017	0.022
9	4	32751	0.498	0.998	0.75	<0.001	0.016	0.007	0.04	0.04	0.037	0.042
10	8	41550	0.491	0.995	0.875	<0.001	0.019	0.012	0.029	0.029	0.027	0.03
11	8	40629	0.488	0.993	0.875	<0.001	0.022	0.014	0.034	0.021	0.02	0.023
12	9	44922	0.49	0.995	0.889	<0.001	0.013	0.008	0.023	0.014	0.013	0.016
13	9	54837	0.497	0.998	0.889	<0.001	0.015	0.007	0.033	0.024	0.022	0.025
14	13	57780	0.489	0.996	0.923	<0.001	0.022	0.015	0.031	0.019	0.018	0.02
15	13	60440	0.492	0.997	0.923	<0.001	0.015	0.01	0.024	0.019	0.018	0.02
16	14	65745	0.495	0.999	0.929	<0.001	0.015	0.008	0.028	0.021	0.02	0.023
17	8	57921	0.496	0.998	0.875	<0.001	0.012	0.006	0.025	0.02	0.018	0.021
18	9	91337	0.493	0.997	0.889	<0.001	0.008	0.004	0.015	0.009	0.008	0.009
19	8	62910	0.497	0.998	0.875	<0.001	0.011	0.005	0.027	0.023	0.021	0.024
20	7	79598	0.496	0.997	0.857	<0.001	0.009	0.004	0.022	0.011	0.01	0.012
21	9	94060	0.494	0.997	0.889	<0.001	0.006	0.003	0.013	0.009	0.008	0.01
22	7	37985	0.496	0.997	0.857	<0.001	0.009	0.002	0.035	0.019	0.016	0.022
23	11	47546	0.492	0.997	0.909	<0.001	0.009	0.004	0.02	0.013	0.012	0.015
24	7	29696	0.482	0.989	0.857	<0.001	0.009	0.005	0.018	0.013	0.012	0.015
25	4	11439	0.249	0.818	0.75	0.176	0.018	0.013	0.023	0.018	0.016	0.021
26	4	12748	0.394	0.896	0.75	0.034	0.019	0.012	0.029	0.019	0.017	0.021
27	4	13899	0.478	0.973	0.75	<0.001	0.006	0.002	0.021	0.005	0.004	0.006
28	3	1045	0.188	0.723	0.667	0.214	0.011	0.005	0.025	0.012	0.007	0.023
29	1	302	NA	NA	NA	NA	0.03	0.016	0.056	0.03	0.016	0.056
30	1	300	NA	NA	NA	NA	0.017	0.007	0.039	0.017	0.007	0.039
31	1	300	NA	NA	NA	NA	0.023	0.011	0.048	0.023	0.011	0.048

The data on the *S*. *japonicum* infection prevalence, using serological methods, in residents in seven endemic provinces (i.e. Anhui, Jiangsu, Jiangxi, Hubei, Hunan, Sichuan and Yunnan) were available from 2003 to 2011 only [2,28,29,30,31,32,33,34,35], which varied between 5.5% (528 089/9 524 813) in 2011 and 8.5% (557 743/6 533 948) in 2004. The pooled seroprevalence in mobile populations in the post-interruption regions each year was significantly lower than the corresponding residents in the endemic regions (see Fig 3), but no significant correlation was observed between both (r_s_ = -0.603, P = 0.086 for a random-effects model, or r_s_ = 0.402, P = 0.284 for a fixed-effects model). No significant and substantial publication bias was found (see Table 4).

**Table 4:** Publication bias of studies examined with Begger test.

		Begger				Begger	
Time	No. studies	Z	P	Years post-interruption	No. studies	Z	P
1992	1	NA		1	1	NA	
1993	1	NA		2	2	1.00	0.32
1994	2	1.00	0.32	3	3	0.52	0.60
1995	5	0.00	1.00	4	2	1.00	0.32
1996	7	-1.05	0.29	5	3	-1.57	0.12
1997	8	0.49	0.62	6	2	1.00	0.32
1998	7	0.19	0.85	7	1	NA	
1999	9	1.04	0.30	8	1	NA	
2000	7	1.65	0.10	9	4	1.36	0.17
2001	8	0.45	0.65	10	8	-0.49	0.62
2002	7	0.75	0.45	11	8	-0.25	0.81
2003	9	0.21	0.84	12	9	0.83	0.40
2004	10	0.80	0.42	13	9	-0.25	0.81
2005	13	0.69	0.49	14	13	1.22	0.22
2006	16	0.63	0.53	15	13	0.61	0.54
2007	14	0.05	0.96	16	14	0.61	0.54
2008	20	0.72	0.47	17	8	0.99	0.32
2009	13	0.41	0.68	18	9	0.25	0.81
2010	9	1.98	0.05	19	8	1.24	0.22
2011	7	1.05	0.29	20	7	0.15	0.88
2012	3	0.52	0.60	21	9	0.74	0.46
2013	1	NA		22	7	1.35	0.18
				23	11	0.45	0.66
				24	7	-0.75	0.45
				25	4	0	1
				26	4	0.68	0.50
				27	4	1.57	0.12
				28	3	1.00	0.32
				29	1	NA	
				30	1	NA	
				31	1	NA	

## Discussion

The results of our meta-analyses indicate that infections with *S*. *japonicum* in mobile populations are unlikely to be the key driving forces causing re-emergence of this disease in previously endemic but now non-endemic (i.e. post interruption) areas. We found that the pooled seroprevalences in the mobile population in China living in post-interruption areas have been low since 1994, and significantly were lower than in residents in areas of China which remain endemic. The estimated seroprevalences in the mobile populations has slowly but significantly decreased over time and with year post-transmission interruption. This is the first time, to our knowledge, that the overall seroprevalence over time in mobile populations in such areas has been estimated.

The serological methods used in surveillance for schistosome infections varied among the included studies. The Kato-Katz stool examination [81] is useful only when the intensity of infection is high and not suitable for post-interruption surveillance due to low sensitivity [82]. The range of serological techniques discussed in the methods differs in their sensitivity, but also in their ability to detect current versus previous infections. As we included studies which had used antibody detection, prevalence measures may have been overestimated in some studies. Results from this meta-analysis show great optimism, as the pooled seroprevalence in mobile populations has been much lower since 1994 or approximately five years after the transmission of the disease had been interrupted, even despite these potential overestimations. Given the fact that more convenient and advanced assays are now applied than before, it may be inferred that the prevalence could have been slightly underestimated in earlier years, indicating that the decreasing trend of the prevalence in the mobile populations over time may be even more pronounced.

Previous research suggested that, due to the influence of environmental and social factors, schistosomiasis had been re-emerging in 38 counties within the same seven Chinese provinces investigated here, where the disease transmission had previously been interrupted or well controlled [4, 24]. It was also reported that an average of ‘return time’ (from control to re-emergence) was about eight years [3]. Therefore, the establishment and implementation of an effective and sustainable surveillance system in the longer term is a great challenge as a country makes the transition from low transmission to elimination of schistosomiasis [83]. The results from our analyses demonstrated that infection prevalence in the mobile population was much lower than that among the corresponding residents in original endemic provinces. When compared with the serological prevalence in local residents, we also found that, out of 13 papers which reported the seroprevalence in the local residents where the populations have migrated into, four showed a lower level of seroprevalence among the local residents than in the mobile population and nine showed a higher or similar level. This indicates that the chance of the source of infection being from the mobile population is currently rare, and potentially greatly over exaggerated. As we observed no substantial publication bias, our findings likely reflect the true seroprevalence, rather than over estimations of the risks associated with mobile populations. This may not have been expected, as often there are more publications of positive findings, which in this instance could influence preconceptions of risks posed by these mobile populations. As no publication bias was observed, an additional explanation for this misconception may be that positive findings were more widely circulated than negative ones, misleading both the general public and policy makers, who may therefore misdirect control measures at these groups, when they may be much better targeted to other potential factors such as animal reservoirs. On the other hand, the decreasing trend of seroprevalence in mobile populations with year post-interruption suggests that consistent and effective control measures have been performed on humans in the areas of their origin and much can be learnt from this.

Transmission of schistosomes is complicated, with about 46 mammals serving as potential reservoirs. Recent studies have shown that in the previously well-controlled areas in Anhui province, China, rodents have become the main reservoirs [84], which has long been ignored, and that dogs could spread the parasite over longer distances [85]. It is highly likely that some potential reservoirs (i.e. rodents, stray dogs or cats, etc.) might contribute greatly to the re-emergence of the disease [86], particularly in areas where snails were found, although further explanations could not be excluded. Once the disease re-emerges in these areas, a top priority should be given to snail control, as schistosomiasis control in wild animal reservoirs is difficult. In addition, more or integrated control measures, as suggested [87], are needed.

Although we indicate that mobile populations present only a small risk to re-emergence, the current surveillance system for infections requires improvement. Firstly, only people coming from endemic areas would require serological detection for schistosomiasis, and if key regions where infections were originating from could be identified which had more specifically been linked to re-emergence in individual regions, then increased control measures in these specific endemic regions may have knock on benefits to reducing re-emergence in other post-interruption areas. Spatial mapping could inform on if risk was associated with geographical distance, versus other factors affecting direction of mobile population movement. Here we observed that, out of 41 included reports, 20 did not document the origin of the mobile people. In the other 21 studies, most reported the provinces rather than the counties the mobile population come from. From the investigation conducted in the suburb of Shanghai City, among 2931 mobile people investigated, 1938 (66.12%) came from *S*. *japonicum*-endemic provinces with its positive rate of 5.99%, significantly higher than those from transmission-interrupted provinces or from non-endemic provinces [60]. Therefore, screening out the ‘high-risk’ people (i.e. from endemic counties) through questionnaires would be helpful in reducing the number of people tested, enabling more intense sampling of those tested and therefore increase the accuracy of prevalence estimates [88,89]. Second, in endemic areas, all residents should receive a serological test for the parasite, and treatment, before leaving the home county for a job elsewhere [90]. Finally, clinical training in schistosomiais diagnosis must be emphasized in both disease endemic and interrupted areas [91], both for the benefit of human health and for the consolidation of control effect. Population genetic analyses often offers insight into transmission networks and gene flow [92], however due to low infection intensities in these infected individuals, direct parasite sampling would be limited.

Limitations in our meta-analyses included that studies often come from well-developed provinces or municipalities, such as Shanghai, Zhejiang and Jiangsu, potentially biasing the impact on the estimated seroprevalence across China. Second, we did not separate active surveillance from passive surveillance [93], as most studies did not have enough related information. Active surveillance could be of more importance in identifying infection sources [93]; whereas passive surveillance could be more useful in finding acute cases or patients with morbidity [90]. Third, a potential bias in the prevalence estimates could exist due to the variation among diagnostic tests, and further work considering the sensitivity and specificity of each test would be highly valuable. Finally, less recent data, for example inform as recent as 1992 onwards [52], were included in this meta-analysis. However, all data were pooled each year in order to show the seroprevalence profile over time.

Although schistosomiasis control was once believed to be a long march [4], the great and exciting achievement obtained across global endemic areas have encouraged experts and policy makers to move ahead—setting the agenda for the elimination of schistosomiasis in China and other countries [94,95]. However, the increasing number of mobile populations may, if some of them are infected with the parasite, raise the worry of re-emergence and transmission of the disease in previously interrupted areas. The results from our systematic review and meta-analysis showed that the seroprevalence in the mobile populations is lower than the residents of the provinces they have moved to and that it is slowly decreasing with time post-interruption. Among 13 eligible research papers, only four reported a higher level of infection in the mobile populations than in the local residents. This suggests that, with the current mobile population levels and current control measures carried out in humans, the chances for *S*. *japonicum* to be spread by the mobile population back into post-interruption areas of China is rare, and that other factors are likely to be more important in the re-emergence of transmission. Nevertheless, a strengthened and innovative surveillance approach is still required if elimination of the disease is to succeed [96], particularly focusing on the origins of infected individuals and where they are moving to, down to the town level, so that policy makers can produce accurate and beneficial guidelines, maximize diagnosis and treatment success and ultimately prevent re-introduction of infections in such areas.

## Supporting Information

S1 Table PRISMA checklist. (DOC)

S2 Table Serological prevalence of *Schistosoma japonicum* in mobile populations and in local populations by study and year. (DOC)

S3 Table Included publications. (XLS)

S4 Table Excluded publications. (XLS)

## References

[1] King CH, Dickman K, Tisch DJ. Reassessment of the cost of chronic helmintic infection: a meta-analysis of disability-related outcomes in endemic schistosomiasis. Lancet. 2005; 365: 1561–1569. 15866310 10.1016/S0140-6736(05)66457-4

[2] Zhen H, Zhang LJ, Zhu R, Xu J, Li SZ, Guo JG, et al Schistosomiasis situation in China in 2011. Chin J Schisto Control. 2012; 24: 621–626. 23593826

[3] Liang S, Yang C, Zhong B, Qiu D. Re-emerging schistosomiasis in hilly and mountainous areas of Sichuan, China. Bulletin of the World Health Organization. 2006; 84: 139–144. 16501732 10.2471/blt.05.025031 PMC2626530

[4] Utzinger J, Zhou XN, Chen MG, Bergquist R. Conquering schistosomiasis in China: the long march. Acta Trop. 2005; 96: 69–96. 16312039 10.1016/j.actatropica.2005.08.004

[5] Zhen J, Guo JG, Zhu HQ. Mobile people and schistosome transmission. Chin J Schisto Control. 1999; 11: 125–127.

[6] Population and Family Planning Commission of China Report on mobile people in China in 2013. Available: http://wwwchinapopgovcn/.

[7] Li SZ, Acosta L, Wang XH, XU LL, Wang Q, Qian YJ, et al Schistosomiasis in China: acute infections during 2005–2008. Chinese Medical Journal. 2009; 122: 1009–1014. 19493433

[8] Leshem E, Meltzer E, Marva E, Schwartz E. Travel-related schistosomiasis acquired in Laos. Emerg Infect Dis. 2009; 15: 1823–1826. 10.3201/eid1511.090611 19891875 PMC2857239

[9] Wang W, Liang YS, Hong QB, Dai JR. African schistosomiasis in mainland China: risk of transmission and countermeasures to tackle the risk. Parasit Vectors. 2013; 6: 249 10.1186/1756-3305-6-249 23985040 PMC3765897

[10] Song HH, Xu HM. Analysis on surveillance of schistosomiasis, malaria and filariasis in the mobile population in Nanhui district of Shanghai for 10 years. Disease surveillance. 2005; 20: 97–98, 101.

[11] Zhang JF, Huang XT, Wen LY, Jiang YH, Zhu MD, Xu GY, et al Surveillance of schistosomiasis in Changshan county in Zhejiang province from 2005 to 2009. Disease surveillance. 2011; 26: 340–343, 354 10.1007/s11606-010-1521-8 20922495 PMC3043176

[12] Lin DD, Liu JX, Liu YM, Hu F, Zhang YY, Xu JM, et al Routine Kato-Katz technique underestimates the prevalence of *Schistosoma japonicum*: a case study in an endemic area of the People's Republic of China. Parasitol Int. 2008; 57: 281–286. 10.1016/j.parint.2008.04.005 18485807

[13] Zhou SF, Yu LX, Wang SP. Progress on the diagnostic approaches for schistosomiasis. J Trop Med. 2009; 9: 335–340.

[14] Song H, Liang Y, Dai J, Ji C, Shen X, Li L, et al Evaluation on dipstick dye immunoassay for screening chemotherapy targets of schistosomiasis in a lower endemic area. Chin J Schisto Cont. 2003; 15: 102–103.

[15] Wang W, Li Y, Li H, Xing Y, Qu G, Dai J, et al Immunodiagnostic efficacy of detection of *Schistosoma japonicum* human infections in China: a meta analysis. Asian Pac J Trop Med. 2012; 5: 15–23. 10.1016/S1995-7645(11)60238-1 22182637

[16] Zhou XN, Xu J, Chen HG, Wang TP, Huang XB, Lin DD, et al Tools to support policy decisions related to treatment strategies and surveillance of *Schistosomiasis japonica* towards elimination. PLoS Negl Trop Dis. 2011; 5: e1408 10.1371/journal.pntd.0001408 22206024 PMC3243709

[17] Li YH, Hu YD, Xu WM, Yang GB, Yin SL, Gong W, et al Comparison of immunoenzymatic staining technique (IEST), double gluing strip circumoval precipitin test (DGS-COPT) and conventional circumoval precipitin test (CV-COPT) for diagnosis of schistosomiasis japonica. Zhongguo Ji Sheng Chong Xue Yu Ji Sheng Chong Bing Za Zhi. 1989; 7: 263–266. 2517415

[18] Zhu Y, He W, Liang Y, Xu M, Yu C, Hua W, et al Development of a rapid, simple dipstick dye immunoassay for schistosomiasis diagnosis. J Immunol Methods. 2002; 266: 1–5. 12133617 10.1016/s0022-1759(02)00086-8

[19] Zhu YC, Socheat D, Bounlu K, Liang YS, Sinuon M, Insisiengmay S, et al Application of dipstick dye immunoassay (DDIA) kit for the diagnosis of schistosomiasis mekongi. Acta Trop. 2005; 96: 137–141. 16143289 10.1016/j.actatropica.2005.07.008

[20] King CH, Bertsch D. Meta-analysis of urine heme dipstick diagnosis of *Schistosoma haematobium* infection, including low-prevalence and previously-treated populations. PLoS Negl Trop Dis. 2013; 7: e2431 10.1371/journal.pntd.0002431 24069486 PMC3772022

[21] Wen LY, Chen JH, Ding JZ, Zhang JF, Lu SH, Yu LL, et al Evaluation on the applied value of the dot immunogold filtration assay (DIGFA) for rapid detection of anti-*Schistosoma japonicum* antibody. Acta Trop. 2005; 96: 142–147. 16207482 10.1016/j.actatropica.2005.07.025

[22] Zhu YC. Immunodiagnosis and its role in schistosomiasis control in China: a review. Acta tropica. 2005; 96: 130–136. 16143288 10.1016/j.actatropica.2005.07.007

[23] Wallace BC, Schmid CH, Lau J, Trikalinos TA. Meta-Analyst: software for meta-analysis of binary, continuous and diagnostic data. BMC Med Res Methodol. 2009; 9: 80 10.1186/1471-2288-9-80 19961608 PMC2795760

[24] Zhou XN, Wang LY, Chen MG, Wu XH, Jiang QW, Chen XY, et al The public health significance and control of schistosomiasis in China--then and now. Acta tropica. 2005; 96: 97–105. 16125655 10.1016/j.actatropica.2005.07.005

[25] Patsopoulos NA, Evangelou E, Ioannidis JP. Heterogeneous views on heterogeneity. Int J Epidemiol. 2009; 38: 1740–1742. 10.1093/ije/dyn235 18940836 PMC4719167

[26] Lau J, Ioannidis JP, Terrin N, Schmid CH, Olkin I. The case of the misleading funnel plot. Bmj. 2006; 333: 597–600. 16974018 10.1136/bmj.333.7568.597 PMC1570006

[27] Ioannidis JP, Trikalinos TA. The appropriateness of asymmetry tests for publication bias in meta-analyses: a large survey. Cmaj. 2007; 176: 1091–1096. 17420491 10.1503/cmaj.060410 PMC1839799

[28] Lei ZL, Zhen H, Zhang LJ, Zhu R, Guo JG, Li SZ, et al Schistosomiasis situation in China in 2010. Chin J Schisto Control. 2011; 23: 599–604. 22379811

[29] Xiao DL, Yu Q, Dang H, Guo JG, Zhou XN, Wang LY. Schistosomiasis situation in China in 2003. Chin J Schisto Control. 2004; 16: 401–404.

[30] Hao Y, Wu XH, Xia G, Zhen H, Guo JG, Wang LY, et al Schistosomiasis situation in China in 2004. Chin J Schisto Control. 2005; 17: 401–404.

[31] Hao Y, Wu XH, Xia G, Zhen H, Guo JG, Wang LY, et al Schistosomiasis situation in China in 2005. Chin J Schisto Control. 2006; 18: 321–324.

[32] Hao Y, Wu XH, Zhen H, Wang LY, Guo JG, Xia G, et al Schistosomiasis situation in China in 2006. Chin J Schisto Control. 2007; 19: 401–404.

[33] Hao Y, Wu XH, Zhen H, Wang LY, Guo JG, Xia G, et al Schistosomiasis situation in China in 2007. Chin J Schisto Control. 2008; 20: 401–404.

[34] Hao Y, Zhen H, Zhu R, Guo JG, Wang LY, Chen Z, et al Schistosomiasis situation in China in 2009. Chin J Schisto Control. 2010; 22: 521–527.

[35] Hao Y, Zhen H, Zhu R, Guo JG, Wu XH, Wang LY, et al Schistosomiasis situation in China in 2008. Chin J Schisto Control. 2009; 21: 451–456.

[36] Moher D, Liberati A, Tetzlaff J, Altman DG. Preferred reporting items for systematic reviews and meta-analyses: the PRISMA statement. Bmj. 2009; 339: b2535 10.1136/bmj.b2535 19622551 PMC2714657

[37] The PLoS Medicine Editors. Best practice in systematic reviews: the importance of protocols and registration. PLoS Med. 2011; 8: e1001009 10.1371/journal.pmed.1001009 21364968 PMC3042995

[38] Handing P, Desheng H, Ketai W. Approach to surveillance and consolidation during past 15 years after elimination of schistosomiasis in Shanghai. Acta Trop. 2002; 82: 301–303. 12020905 10.1016/s0001-706x(02)00023-2

[39] Wu WL, Wang JR, Wen LY, Huang YY, Xu XF, Yu WM. Surveillance and control of post-transmission schistosomiasis in Jiaxing prefecture, Zhejiang province, China. Acta Trop. 2005; 96: 282–287. 16198300 10.1016/j.actatropica.2005.07.022

[40] Wu XH, Chen MG, Zheng J. Surveillance of schistosomiasis in five provinces of China which have reached the national criteria for elimination of the disease. Acta Trop. 2005; 96: 276–281. 16198301 10.1016/j.actatropica.2005.07.021

[41] Sleigh A, Li X, Jackson S, Huang K. Eradication of schistosomiasis in Guangxi, China. Part 1: Setting, strategies, operations, and outcomes, 1953–92. Bull World Health Organ. 1998; 76: 361–372. 9803587 PMC2305772

[42] Fang YM, Cheng YF, Fang RL, Hu ZY, Wang RB, Zhu JM, et al Impact of mobile populations on transmission of schistosomiasis in transmission-interrupted areas. Chin J Schisto Control. 2009; 121: 553–554.

[43] Yan CL, Lin GH, Guo XK, Huang MS. Surveillance of schistosomiasis during 1989 to 2010 in Longhai city. Strait J Prev Med. 2012; 18: 52–53.

[44] Xu CH, Pan ZM, Liu XN, Ren WF, Guo RT, Zhong F. Surveillance of schistosomiasis in Guangzhou during 2006 to 2010. Disease surveillance. 2013; 28: 389–391.

[45] Li CS, Zhou ZS, Huang DH, Kuang XJ, Huang SY. Endemic situation of schistosomiasis in Qingxin county, Guangdong from 2006 to 2010. J Trop Med. 2012; 12: 348–351.

[46] Gao YF, Ren WF, Gao RT. Analysis of the monitoring on schistosomiasis in the endemic area of Guangzhou from 1996 to 2000. J Trop Med. 2003; 3: 87–88, 84.

[47] Gao YF, Ren WF, Guo RT, Feng YJ, Liu XN, Pan ZM. Analysis of the monitoring on schistosomiasis in the endemic area of Guangzhou from 2001 to 2005. J Trop Med. 2007; 7: 927–928.

[48] Zhou XH, Chen LG, Luo JP, He LJ, Pan D, Cui WJ. Results of the monitoring of schistosmiasis in Shaoguan city from 2001 to 2006. Chin Trop Med. 2008; 8: 624–625.

[49] Su JH. Surveillance of schistosomiasis in Gaoxing district of Zhaoqing city from 2008–2013. Jiangsu J Prev Med. 2014; 25: 68–69.

[50] Lin R, Li XM, Zhang HM, Tan YG, Zhang LJ, Huang FM, et al Analysis of shistosomiasis surveillance in mobile populations in Guangxi in 2008. Chin J Schisto Control. 2009; 21: 528–531.

[51] Zhou WE. The monitoring of schistosomiasis in the provincial monitoring points within Changshu city in 2008. Occup and Health. 2009; 25: 1628–1629.

[52] Wu YZ, Zhen XY, Sun GX, Wu GQ, Chen JJ, Chen JY, et al Investigation of schistosomiasis in mobile populations with PVC membrane. Chin J Parasitol & Dis. 1996; 14: 160 10.1645/GE-1250.1 22347291 PMC3279879

[53] Xue CL, Rong RQ, Wang SZ. Investigation of schistosomiasis, filariasis and malaria in mobile populations. Prev Med Lit Inf. 1997; 3: 116.

[54] Zhou YJ. Analysis on surveillance results of schistosomiasis in Sian township of Nantong city from 2002 to 2009. Occup & Health. 2011; 27: 1011–1012.

[55] Ji P. Report on surveillance of schistosomiasis in Tongzhou city from 1996 to 1999. Chinese Primary Health Care. 2000; 14: 32.

[56] Jin YJ, Cai L, Yang J, Wang HX, Yu RF, Liu GG, et al Surveillance on schistosomiasis in Shanghai in 2005. Journal of Tropical Diseases and Parasitology. 2006; 4: 220–221, 228.

[57] Yang J, Wang HX, Jin YJ, Cai L, Yu RF. Report on surveillance of schistosomiasis in Jinshan district in Shanghai in 2006. Journal of Tropical Diseases and Parasitology. 2007; 5: 240–241.

[58] Yang J, Wang HX, Jin YJ, Cai L, Yu RF, Zhou PP, et al Analysis of surveillance results of schistosomiasis in Jinshan district in Shanghai in 2007. Journal of Tropical Diseases and Parasitology. 2008; 6: 223–224.

[59] Yu RF, Wang HX, Zhou PP. Report on surveillance of schistosomiasis in Jinshan district in Shanghai in 2008. Journal of Tropical Diseases and Parasitology. 2009; 7: 229–230.

[60] Zhou XN, Cai L, Zhang XP, Sheng HF, Ma XB, Jin YJ, et al Potential risks for transmission of schistosomiasis caused by mobile population in Shanghai. Chin J Parasit Dis Con. 2007; 25: 180–184. 18038772

[61] Shi WP, Shen HG, Wang WY, Su HL, Zhao DX, Yu LP. Analysis on surveillance of malaria, schistosomiasis and filariasis in Minhang district of Shanghai from 1994 to 2009. Modern Preventive Medicine. 2012; 39: 1236–1237, 1243.

[62] He TC, Wang HX. The components of the mobile population and its prevalence of malaria, schistosomiasis and filariasis in Jinshan district of Shanghai in 1999. Sh J Prev Med. 2002; 14: 144.

[63] Song HH. Analysis on surveillance of malaria, schistosomiasis and filariasis in the mobile population in Nanhui district of Shanghai for 10 years. China Tropical Medicine. 2011; 11: 1344–1345.

[64] Jin YJ, Cai L, Sun CY, Wang HX, Jiang PH, He YY, et al Study on the seroepidemiology of schistosomiasis in the mobile population in Shanghai. Journal of Tropical Medicine. 2010; 10: 999–1002.

[65] Qiu XF, Lu L, Zhang ZX, Qi JP. Analysis on surveillance of schistosomiasis, malaria and filariasis in the mobile population in Luwan district of Shanghai. Sh J Prev Med. 2010; 22: 215–216.

[66] He F, Yu XQ. Consolidation and Surveillance on schistosomiasis in Qinpu district of Shanghai since transmission interrupted. Sh J Prev Med. 2006; 18: 399–400.

[67] Dang H, Guo JG, Wang Q, Yu Q, Zhu HQ, Xu J, et al Investigation on potential risks of schistosome transmission among immigrants in Shanghai. Chin J Schisto Control. 2005; 17: 383–384.

[68] Li YF. Surveillance on schistosomiasis, malaria and filariasis in the mobile population in Baoshan district of Shanghai. Sh J Prev Med. 1996; 8: 252–253.

[69] Yuan GP, Gu MM, Li WJ, Meng Y, Zhang YJ. Surveillance on schistosomiasis, malaria and filaraisis in the immigrants in Baoshan district of Shanghai in 2000. Occup & Health. 2002; 18: 58–59.

[70] Li JL, Chen HY, Feng TC. Surveillance of schistosomiasis in the mobile population in Linping district of Hangzhou city in 2005. Zhejiang Prev Med. 2007; 19: 35.

[71] Xie JR, Chen ZH, Yu LY. Surveillance of schistosomiasis in the mobile population in Zhuji city from 2008 to 2009. Chin J Schisto Control. 2010; 22: 514.

[72] Zhu PH, Xu HQ. Surveillance of schistosomiasis in rivers and lake areas in Jiaxing, Zhejiang from 2008 to 2011. Disease surveillance. 2012; 27: 650–653.

[73] Chen LQ, Shi NF, Fan FN, Fang GY, Yu JF. Surveillance of schistosomiasis in the mobile population in Cixi city of Zhejiang province from 2004 to 2007. Disease surveillance. 2008; 23: 322–323.

[74] Xu JY, Chen GH, Fan FN. Surveillance of schistosomiasis since disease interrupted. Chin J Vector Bio & Control. 2009; 20: 351.

[75] Xu QH, Zhang JF, Jiang YH. Analysis on schistosomiasis in the surveillance site in Changshan county from 2008 to 2011. Chin J of PHM. 2012; 28: 634–636.

[76] Wang JR, Wu YK, Cao NX, Zhang HF, Zhu PH, Ma QQ, et al Surveillance of schistosomiasis since transmission interrupted in Jiaxing city, Zhejiang province from 1995 to 2012. Disease surveillance. 2013; 28: 567–569.

[77] Zhou AF, Wang JR, Yu WM, Shen JY. Surveillance of schistosomiasis, malaria and filariasis in the mobile population in Jiaxing city. Zhejiang Prev Med. 1998; 7: 391–392. 9610788

[78] Xu LR, Shen P, Lu HC, Zhou LS, Chen Y, Zhou BB, et al Serological surveillance of infectious diseases in the mobile population in Jinzhou district of Ningbo city Chin Prev Med. 2009; 10: 44–48.

[79] Lou J, Lou JY, Chen JS, Wang ZH. The application of Dot Immuno-Gold Filtration Assay on surveillance of schistosomiasis in the mobile population. Zhejiang Prev Med. 2001; 13:17.

[80] Hu CY, Fan FN. Surveillance of schistosomiasis in Cixi city from 2008 to 2012. Zhejiang Prev Med. 2014; 26: 280–282.

[81] Katz N, Chaves A, Pellegrino J. A simple device for quantitative stool thick-smear technique in Schistosomiasis mansoni. Journal of the São Paulo Institute of Tropical Medicine. 1972; 14: 397–400. 4675644

[82] Wu G. A historical perspective on the immunodiagnosis of schistosomiasis in China. Acta Trop. 2002; 82: 193–198. 12020892 10.1016/s0001-706x(02)00010-4

[83] Spear RC, Seto EY, Carlton EJ, Liang S, Remais JV, Zhong B, et al The challenge of effective surveillance in moving from low transmission to elimination of schistosomiasis in China. Int J Parasitol. 2011; 41: 1243–1247. 10.1016/j.ijpara.2011.08.002 21920366 PMC3191863

[84] Lu DB, Rudge JW, Wang TP, Donnelly CA, Fang GR, Webster JP. Transmission of *Schistosoma japonicum* in marshland and hilly regions of China: parasite population genetic and sibship structure. PLoS Negl Trop Dis. 2010; 4: e781 10.1371/journal.pntd.0000781 20689829 PMC2914789

[85] Rudge JW, Webster JP, Lu DB, Wang TP, Fang GR, Basanez MG. Identifying host species driving transmission of schistosomiasis japonica, a multihost parasite system, in China. Proc Natl Acad Sci USA. 2013; 110: 11457–11462. 10.1073/pnas.1221509110 23798418 PMC3710859

[86] Lu DB, Wang TP, Rudge JW, Donnelly CA, Fang GR, Webster JP. Contrasting reservoirs for *Schistosoma japonicum* between marshland and hilly regions in Anhui, China—a two-year longitudinal parasitological survey. Parasitology. 2010; 137: 99–110. 10.1017/S003118200999103X 19723358

[87] Wang LD, Chen HG, Guo JG, Zeng XJ, Hong XL, Xiong JJ, et al A strategy to control transmission of *Schistosoma japonicum* in China. New England Journal of Medicine. 2009; 360: 121–128. 10.1056/NEJMoa0800135 19129526

[88] Li JH, Wang TP, Xiao X, Wu WD, Lu DB, Fang GR, et al Cost-effectiveness analysis on different screening methods for schistosomiasis in hypo-endemic areas. Practical Prevention of Parasitic Diseases. 2002; 10: 145–148.

[89] Lin LJ, Wen LY, Zhu MD, Yan XL, Chen W, Zhang JF, et al Sampling survey on the prevalence of schistosomiasis among floating population in Zhejiang province. Chin J Clin Infect Dis. 2010; 3: 340–342, 371.

[90] Yu Q, Zhao GM, Cao CL, Huang SY, Zhang HM, Zhang JF, et al Cost-effectiveness analysis on screening for surveillance of schistosomiasis in population in transmission interrupted areas. Chin J Schisto Control. 2007; 19: 46–49.

[91] Lu DB, Zhou L, Li Y. Improving access to anti-schistosome treatment and care in nonendemic areas of china: lessons from one case of advanced schistosomiasis japonica. PLoS Negl Trop Dis. 2013; 7: e1960 10.1371/journal.pntd.0001960 23349997 PMC3547857

[92] Gower CM, Gouvras AN, Lamberton PH, Deol A, Shrivastava J, Mutombo PN, et al Population genetic structure of *Schistosoma mansoni* and Schistosoma haematobium from across six sub-Saharan African countries: Implications for epidemiology, evolution and control. Acta Trop. 2013; 128: 261–274. 10.1016/j.actatropica.2012.09.014 23041540

[93] Li L, Wen LY. Epidemic and control of schistosomiasis in migrant population. Acta Parasitol Med Entomol Sin. 2013; 20: 59–65.

[94] Rollinson D, Knopp S, Levitz S, Stothard JR, Tchuem Tchuente LA, Garba A, et al Time to set the agenda for schistosomiasis elimination. Acta Trop. 2013; 128: 423–440. 10.1016/j.actatropica.2012.04.013 22580511

[95] King CH. Toward the elimination of schistosomiasis. N Engl J Med. 2009; 360: 106–109. 10.1056/NEJMp0808041 19129524

[96] Bergquist R, Tanner M. Controlling schistosomiasis in Southeast Asia: a tale of two countries. Adv Parasitol. 2010; 72: 109–144. 10.1016/S0065-308X(10)72005-4 20624530

