# Supplementary material for: Serological Prevalence of Schistosoma japonicum in Mobile Populations in Previously Endemic but Now Non-Endemic Regions of China: A Systematic Review and Meta-Analysis
Source: PLoS One. 2015 Jun 4;10(6):e0128896. doi: 10.1371/journal.pone.0128896 (PMC4456376; doi:10.1371/journal.pone.0128896)
Supplement: S2 Table — (DOC) [file pone.0128896.s002.doc]

**S2 Table. Serological prevalence (%, no. positive/no. examined) of *Schistosoma japonicum* in the mobile population and in the local population by study and year**

|  |  | Mobile populations | | | Local populations | | |
| --- | --- | --- | --- | --- | --- | --- | --- |
| Author, year | Year survey performed | Prevalence (%) | No. Positive | No. Examined | Prevalence (%) | No. Positive | No. Examined |
| Fang et al, 2009 | 2007 | 0.47 | 2 | 422 | 0.25 | 9 | 3576 |
| Yan et al, 2012 | 2001 | 0 | 0 | 102 | 3.4 | 11 | 328 |
|  | 2008 | 0 | 0 | 410 | 0 | 0 | 41 |
| Xu et al, 2013 | 2006 | 1.50 | 3 | 200 | 0 | 0 | 200 |
|  | 2007 | 1.07 | 11 | 1027 | 0 | 0 | 200 |
|  | 2008 | 3.17 | 19 | 600 | 0 | 0 | 600 |
|  | 2009 | 0.00 | 0 | 300 | 0 | 0 | 300 |
|  | 2010 | 0.00 | 0 | 300 | 0 | 0 | 450 |
| Li et al, 2012 | 2006 | 6.19 | 62 | 1002 | 4.19 | 9 | 215 |
|  | 2007 | 1.00 | 1 | 100 | NA |  |  |
|  | 2008 | 0.93 | 7 | 754 | 0 | 0 | 300 |
|  | 2009 | 2.67 | 8 | 300 | 0.33 | 1 | 307 |
|  | 2010 | 0.67 | 2 | 300 | 0 | 0 | 300 |
| Gao et al, 2003 | 1996 | 4.84 | 3 | 62 | 3.1 | 12 | 393 |
|  | 1997 | 2.22 | 1 | 45 | 2.5 | 9 | 366 |
|  | 1998 | 4.60 | 4 | 87 | 2.3 | 8 | 353 |
|  | 1999 | 5.88 | 6 | 102 | 3.7 | 14 | 375 |
|  | 2000 | 5.08 | 3 | 59 | 4.8 | 18 | 369 |
| Gao et al, 2007 | 2001 | 5.66 | 3 | 53 | 0 | 0 | 208 |
|  | 2002 | 2.08 | 1 | 48 | 0 | 0 | 204 |
|  | 2003 | 4.49 | 4 | 89 | 0 | 0 | 209 |
|  | 2004 | 5.88 | 6 | 102 | 0 | 0 | 200 |
|  | 2005 | 4.69 | 3 | 64 | 0 | 0 | 200 |
| Zhou et al, 2008 | 2001 | 4.11 | 3 | 73 | 0 | 0 | 487 |
|  | 2002 | 2.30 | 2 | 87 | 0 | 0 | 466 |
|  | 2003 | 4.08 | 4 | 98 | 0 | 0 | 428 |
|  | 2004 | 4.76 | 3 | 63 | 0 | 0 | 403 |
|  | 2005 | 5.88 | 3 | 51 | 0 | 0 | 398 |
|  | 2006 | 4.94 | 4 | 81 | 0 | 0 | 387 |
| Su et al, 2014 | 2008 | 1.84 | 8 | 435 | 0.49 | 2 | 412 |
|  | 2009 | 1.56 | 6 | 383 | 0.25 | 1 | 396 |
|  | 2010 | 1.76 | 9 | 512 | 0.61 | 3 | 489 |
|  | 2011 | 1.16 | 4 | 345 | 0.56 | 2 | 358 |
|  | 2012 | 1.71 | 13 | 762 | 0.50 | 3 | 595 |
|  | 2013 | 1.58 | 7 | 443 | 0.32 | 1 | 313 |
| Lin et al, 2009 | 2008 | 3.46 | 84 | 2428 | NA |  |  |
| Zhou et al, 2009 | 2008 | 0.00 | 0 | 124 | 0.47 | 5 | 1066 |
| Wu et al, 1994 | 1992 | 6.87 | 670 | 9759 | 0.1 | 23 | 21421 |
| Xue et al, 1997 | 1995 | 3.75 | 40 | 1067 | NA |  |  |
| Zhou et al, 2011 | 2003 | 0.94 | 2 | 212 | NA |  |  |
|  | 2004 | 1.45 | 3 | 207 | 0.49 | 1 | 205 |
|  | 2005 | 0.79 | 2 | 254 | NA |  |  |
|  | 2006 | 1.08 | 3 | 277 | 0.50 | 1 | 200 |
|  | 2007 | 1.44 | 3 | 209 | NA |  |  |
|  | 2008 | 1.20 | 3 | 249 | NA |  |  |
|  | 2009 | 0.64 | 2 | 312 | NA |  |  |
| Ji et al, 2000 | 1996 | 1.35 | 6 | 446 | NA |  |  |
|  | 1997 | 2.21 | 6 | 272 | 0.85 | 4 | 468 |
|  | 1998 | 0.95 | 3 | 317 | 0.86 | 4 | 464 |
|  | 1999 | 5.65 | 14 | 248 | 1.81 | 6 | 332 |
| Jin et al, 2006 | 2005 | 0.00 | 0 | 103 | 0.59 | 3 | 507 |
|  | 2005 | 0.06 | 7 | 11690 | NA |  |  |
| Yang et al, 2007 | 2006 | 3.56 | 18 | 505 | 4.47 | 23 | 514 |
|  | 2006 | 0.06 | 8 | 12747 | NA |  |  |
| Yang et al, 2008 | 2007 | 0.60 | 3 | 504 | 1 | 5 | 501 |
|  | 2007 | 0.05 | 7 | 14300 | NA |  |  |
| Yu et al, 2009 | 2008 | 1.66 | 10 | 602 | NA |  |  |
| Zhou et al, 2007 | 2004 | 4.71 | 138 | 2931 | NA |  |  |
| Shi et al, 2012 | 1994 | 5.37 | 447 | 8325 | NA |  |  |
|  | 1995 | 3.94 | 549 | 13491 | NA |  |  |
|  | 1996 | 1.72 | 251 | 14588 | NA |  |  |
|  | 1997 | 0.71 | 108 | 15268 | NA |  |  |
|  | 1998 | 0.39 | 60 | 15201 | NA |  |  |
|  | 1999 | 1.21 | 134 | 11077 | NA |  |  |
|  | 2000 | 0.62 | 53 | 8581 | NA |  |  |
|  | 2001 | 0.31 | 29 | 9467 | NA |  |  |
|  | 2002 | 0.34 | 47 | 13963 | NA |  |  |
|  | 2003 | 0.22 | 81 | 37393 | NA |  |  |
|  | 2004 | 0.21 | 57 | 27008 | NA |  |  |
|  | 2005 | 0.17 | 53 | 30823 | NA |  |  |
|  | 2006 | 0.10 | 37 | 35995 | NA |  |  |
|  | 2007 | 0.05 | 9 | 18964 | NA |  |  |
|  | 2008 | 0.09 | 20 | 22509 | NA |  |  |
|  | 2009 | 0.13 | 13 | 10791 | NA |  |  |
| He et al, 2002 | 1999 | 4.45 | 57 | 1281 | NA |  |  |
| Song et al, 2011 | 2000 | 0.36 | 22 | 6190 | NA |  |  |
|  | 2001 | 0.13 | 8 | 5986 | NA |  |  |
|  | 2002 | 0.18 | 12 | 6810 | NA |  |  |
|  | 2003 | 0.40 | 34 | 8487 | NA |  |  |
|  | 2004 | 0.54 | 43 | 7971 | NA |  |  |
|  | 2005 | 0.52 | 57 | 11019 | NA |  |  |
|  | 2006 | 0.44 | 26 | 5957 | NA |  |  |
|  | 2007 | 0.49 | 19 | 3910 | NA |  |  |
|  | 2008 | 1.00 | 47 | 4723 | NA |  |  |
|  | 2009 | 1.01 | 37 | 3671 | NA |  |  |
| Jin et al, 2010 | 2008 | 2.14 | 64 | 2992 | NA |  |  |
| Qiu et al, 2010 | 1995 | 2.02 | 64 | 3168 | NA |  |  |
|  | 1996 | 1.44 | 43 | 2977 | NA |  |  |
|  | 1997 | 1.79 | 83 | 4635 | NA |  |  |
|  | 1998 | 1.25 | 39 | 3115 | NA |  |  |
|  | 1999 | 1.21 | 47 | 3879 | NA |  |  |
|  | 2000 | 0.89 | 39 | 4364 | NA |  |  |
|  | 2001 | 1.23 | 40 | 3261 | NA |  |  |
|  | 2002 | 0.43 | 11 | 2561 | NA |  |  |
|  | 2003 | 0.90 | 22 | 2457 | NA |  |  |
|  | 2004 | 0.15 | 5 | 3433 | NA |  |  |
|  | 2005 | 0.10 | 1 | 1001 | NA |  |  |
|  | 2006 | 0.41 | 3 | 725 | NA |  |  |
|  | 2007 | 1.46 | 11 | 755 | NA |  |  |
|  | 2008 | 2.32 | 19 | 819 | NA |  |  |
| Song et al, 2005 | 1994 | 0.17 | 10 | 6060 | NA |  |  |
|  | 1995 | 0.22 | 13 | 5938 | NA |  |  |
|  | 1996 | 0.05 | 2 | 4177 | NA |  |  |
|  | 1997 | 0.20 | 9 | 4429 | NA |  |  |
|  | 1998 | 0.00 | 0 | 3622 | NA |  |  |
|  | 1999 | 0.67 | 18 | 2687 | NA |  |  |
| He et al, 2006 | 1996 | 2.88 | 151 | 5247 | NA |  |  |
|  | 1997 | 2.10 | 195 | 9267 | NA |  |  |
|  | 1998 | 3.52 | 291 | 8267 | NA |  |  |
|  | 1999 | 3.47 | 498 | 14332 | NA |  |  |
|  | 2000 | 2.85 | 518 | 18181 | NA |  |  |
|  | 2001 | 1.44 | 337 | 23434 | NA |  |  |
|  | 2002 | 2.74 | 579 | 21138 | NA |  |  |
|  | 2003 | 1.20 | 410 | 34228 | NA |  |  |
|  | 2004 | 1.48 | 297 | 20017 | NA |  |  |
| Dang et al, 2005 | 2003 | 0.32 | 1 | 313 | NA |  |  |
| Li et al, 1996 | 1993 | 5.61 | 270 | 4809 | NA |  |  |
| Yuan et al, 2002 | 2000 | 0.56 | 27 | 4797 | NA |  |  |
| Zhang et al, 2011 | 2005 | 5.83 | 6 | 103 | 4.91 | 26 | 530 |
|  | 2006 | 3.81 | 4 | 105 | 3.77 | 19 | 504 |
|  | 2007 | 6.48 | 7 | 108 | 8.12 | 41 | 505 |
|  | 2008 | 0.94 | 1 | 106 | 6.79 | 34 | 501 |
|  | 2009 | 0.85 | 1 | 118 | 0.20 | 1 | 504 |
|  | 2005 | 1.67 | 15 | 896 | NA |  |  |
|  | 2006 | 3.64 | 28 | 769 | NA |  |  |
|  | 2007 | 5.01 | 38 | 758 | NA |  |  |
|  | 2008 | 1.26 | 5 | 396 | NA |  |  |
|  | 2009 | 1.98 | 5 | 252 | NA |  |  |
|  | 2005 | 3.44 | 21 | 610 | NA |  |  |
|  | 2006 | 11.76 | 32 | 272 | NA |  |  |
|  | 2007 | 3.45 | 5 | 145 | NA |  |  |
|  | 2008 | 21.97 | 87 | 396 | NA |  |  |
|  | 2009 | 13.85 | 32 | 231 | NA |  |  |
| Li et al, 2007 | 2006 | 0.33 | 11 | 3307 | NA |  |  |
| Xie et al, 2010 | 2008 | 5.33 | 48 | 900 | NA |  |  |
|  | 2009 | 3.08 | 61 | 1979 | NA |  |  |
| Zhu et al, 2012 | 2008 | 0.55 | 4 | 723 | 1.14 | NA | NA |
|  | 2009 | 6.70 | 63 | 941 | 5.07 | NA | NA |
|  | 2010 | 5.26 | 43 | 818 | 2.40 | NA | NA |
|  | 2011 | 0.16 | 4 | 2443 | 0.48 | NA | NA |
| Chen et al, 2008 | 2004 | 5.22 | 36 | 690 | NA |  |  |
|  | 2005 | 1.60 | 213 | 13295 | NA |  |  |
|  | 2006 | 1.88 | 180 | 9600 | NA |  |  |
|  | 2007 | 1.83 | 210 | 11503 | NA |  |  |
| Xu et al, 2009 | 2008 | 0.26 | 32 | 12330 | NA |  |  |
| Hu et al, 2014 | 2008 | 0.99 | 5 | 507 | 0.00 | 0 | 281 |
|  | 2009 | 0.99 | 3 | 302 | 0.34 | 1 | 296 |
|  | 2010 | 2.98 | 9 | 302 | 0.47 | 1 | 215 |
|  | 2011 | 1.67 | 5 | 300 | 0.86 | 2 | 232 |
|  | 2012 | 2.33 | 7 | 300 | 0.50 | 1 | 200 |
| Xu et al, 2012 | 2010 | 0.81 | 1 | 124 | 4.79 | 24 | 501 |
|  | 2011 | 3.30 | 7 | 212 | 1.21 | 4 | 330 |
|  | 2010 | 1.63 | 5 | 307 | NA |  |  |
|  | 2011 | 1.87 | 10 | 536 | NA |  |  |
|  | 2010 | 5.36 | 15 | 280 | NA |  |  |
|  | 2011 | 10.00 | 13 | 130 | NA |  |  |
| Wang et al, 2013 | 1995 | 4.85 | 971 | 20005 | 2.24 | 1124 | 50257 |
|  | 1996 | 2.30 | 505 | 21943 | 2.22 | 993 | 44770 |
|  | 1997 | 2.13 | 333 | 15639 | 2.62 | 1105 | 42109 |
|  | 1998 | 2.10 | 259 | 12333 | 2.52 | 745 | 29515 |
|  | 1999 | 1.84 | 235 | 12755 | 2.26 | 546 | 24108 |
|  | 2000 | 1.93 | 205 | 10625 | 0.68 | 69 | 10201 |
|  | 2001 | 1.98 | 179 | 9063 | 0.63 | 77 | 12296 |
|  | 2002 | 1.95 | 210 | 10753 | 0.96 | 92 | 9552 |
|  | 2003 | 1.08 | 147 | 13557 | 1.26 | 116 | 9179 |
|  | 2004 | 2.05 | 342 | 16682 | 1.81 | 155 | 8578 |
|  | 2005 | 2.19 | 384 | 17534 | 2.30 | 106 | 4605 |
|  | 2006 | 1.61 | 258 | 16018 | 0.60 | 15 | 2509 |
|  | 2007 | 2.13 | 564 | 26477 | 1.55 | 40 | 2586 |
|  | 2008 | 1.64 | 452 | 27609 | 1.34 | 29 | 2164 |
|  | 2009 | 1.32 | 370 | 28050 | 1.12 | 24 | 2152 |
|  | 2010 | 0.73 | 187 | 25509 | 1.63 | 32 | 1964 |
|  | 2011 | 0.91 | 140 | 15399 | 0.21 | 2 | 940 |
|  | 2012 | 0.67 | 112 | 16842 | 1.41 | 4 | 283 |
| Zhou et al, 1998 | 1997 | 5.24 | 176 | 3361 | NA |  |  |
| Xu et al, 2009 | 2006 | 0.73 | 142 | 19403 | NA |  |  |
| Lou et al, 2001 | 1999 | 1.88 | 16 | 849 | NA |  |  |
